# Acute Poisoning with Rhabdomyolysis in the Intensive Care Unit: Risk Factors for Acute Kidney Injury and Renal Replacement Therapy Requirement

**DOI:** 10.3390/toxics8040079

**Published:** 2020-09-28

**Authors:** Pierre-François Rogliano, Sebastian Voicu, Laurence Labat, Nicolas Deye, Isabelle Malissin, Jean-Louis Laplanche, Dominique Vodovar, Bruno Mégarbane

**Affiliations:** 1 Department of Medical and Toxicological Critical Care, Federation of Toxicology APHP, Lariboisière Hospital, University of Paris, 75010 Paris, France; pf.rogliano@gmail.com (P.-F.R.); sebastian.voicu@aphp.fr (S.V.); nicolas.deye@aphp.fr (N.D.); isabelle.malissin@aphp.fr (I.M.); dominique.vodovar@aphp.fr (D.V.); 2 Inserm UMRS 1144, University of Paris, 75010 Paris, France; laurence.labat@aphp.fr (L.L.); jean-louis.laplanche@aphp.fr (J.-L.L.); 3 Laboratory of Toxicology, Federation of Toxicology APHP, Lariboisière Hospital, University of Paris, 75010 Paris, France; 4 Laboratory of Biochemistry, Federation of Toxicology APHP, Lariboisière Hospital, University of Paris, 75010 Paris, France

**Keywords:** acute kidney injury, poisoning, predictive factor, renal replacement therapy, rhabdomyolysis

## Abstract

Acute kidney injury (AKI) is the major complication of rhabdomyolysis. We aimed to identify the predictive factors for AKI and renal replacement therapy (RRT) requirement in poisoning-associated rhabdomyolysis. We conducted a cohort study including 273 successive poisoned patients (median age, 41 years) who developed rhabdomyolysis defined as creatine kinase (CK) >1000 IU/L. Factors associated with AKI and RRT requirement were identified using multivariate analyses. Poisonings mainly involved psychotropic drugs. AKI occurred in 88 patients (37%) including 43 patients (49%) who required RRT. Peak serum creatinine and CK were weakly correlated (*R*^2^ = 0.17, *p* < 0.001). Death (13%) was more frequent after AKI onset (32% vs. 2%, *p* < 0.001). On admission, lithium overdose (OR, 44.4 (5.3–371.5)), serum calcium ≤2.1 mmol/L (OR, 14.3 (2.04–112.4)), female gender (OR, 5.5 (1.8–16.9)), serum phosphate ≥1.5 mmol/L (OR, 2.0 (1.0–4.2)), lactate ≥ 3.3 mmol/L (OR, 1.2 (1.1–1.4)), serum creatinine ≥ 125 µmol/L (OR, 1.05 (1.03–1.06)) and age (OR, 1.04 (1.01–1.07)) independently predicted AKI onset. Calcium-channel blocker overdose (OR, 14.2 (3.8–53.6)), serum phosphate ≥ 2.3 mmol/L (OR, 1.6 (1.1–2.6)), Glasgow score ≤ 5 (OR, 1.12; (1.02–1.25)), prothrombin index ≤ 71% (OR, 1.03; (1.01–1.05)) and serum creatinine ≥ 125 µmol/L (OR, 1.01; (1.00–1.01)) independently predicted RRT requirement. We identified the predictive factors for AKI and RRT requirement on admission to improve management in poisoned patients presenting rhabdomyolysis.

## 1. Introduction

Rhabdomyolysis is commonly reported in the poisoned patient with variable consequences ranging from a simple increase in serum creatine kinase (CK) to life-threatening electrolyte disturbances and acute kidney injury (AKI) requiring renal replacement therapy (RRT) [1,2]. In patients presenting rhabdomyolysis, a 13-50% risk of AKI [1,2,3,4] and up to 83% risk of mortality [5] have been reported.

Mechanisms by which toxicants cause rhabdomyolysis are variable including prolonged unconsciousness and immobility, agitation, seizures, fall, withdrawal and hyperthermia [1,2]. Rarely, toxicant-induced direct effects (e.g., with cholesterol-lowering drugs of the statin class or with hair dye containing paraphenylenediamine) or nonspecific alterations in muscle ion homeostasis (e.g., binge drinking ethanol) are hypothesized. Rhabdomyolysis as a result of exposure to natural toxins is also often encountered after eating certain fish or mushroom species or being bitten by a snake or insect. Nutritional deficiencies, hypophosphatemia and hypokalemia may represent coexistent risk factors for the development of rhabdomyolysis [2].

Risk factors for AKI in the presence of rhabdomyolysis include hyperkalemia, hyperphosphatemia, hypocalcemia, dehydration, acidosis, sepsis, intravascular volume depletion, high serum myoglobin concentrations and low myoglobin clearance [1,2]. In the poisoned patient, markers predictive of rhabdomyolysis-related renal complications have been poorly investigated despite the importance of being able to identify patients at risk to whom administering appropriate fluid rehydration with the goal of urine output of 300 mL/h is recommended [2]. Serum CK level is routinely used as marker of severity although its correlation with the onset of consequent renal complications is rather weak [1,2].

We, therefore, designed a study aiming to analyze (1) the drugs involved; (2) the complications observed; and (3) the predictive factors for AKI, RRT requirement and death in those poisoned patients admitted to the intensive care (ICU) who developed rhabdomyolysis.

## 2. Materials and Methods

### 2.1. Study Design and Setting

We conducted a single-center retrospective observational study. Our Hospital ICU is dedicated to poisoned patients, accounting for ~30% of the total admissions. The study was conducted according to the Helsinki principles and approved by our institutional review board (*Commission Nationale de l’Informatique et des Libertés*, N 2067659; date of approval, 26 May 2017).

All poisoned patients admitted to the ICU between January 2012 and June 2018 and who developed rhabdomyolysis were included. Patients with preexisting myositis, myopathy, muscular dystrophy and CK elevation attributed to acute cardiac involvement or chronic renal failure were not included.

Diagnosis of poisoning was made based on history, clinical findings and laboratory confirmations, when available. Patients were managed according to the national guidelines established by the French Intensive Care Society [6]. Fluid management during the first 24 h to compensate dehydration or fluid losses before admission was monitored using ultrasound measurement of inferior vena cava diameter when required by the clinician in charge.

### 2.2. Parameter Definitions

Rhabdomyolysis was considered if at least one serum CK concentration ≥ 1000 IU/L (i.e., ~5 times the upper-limit of normal) was identified during the ICU stay, as generally recognized [2,7,8]. AKI was defined according to the Kidney Disease Improving Global Outcomes (KDIGO) criteria [9]. AKI was classified as “stage 1” if a 1.5 to 1.9-fold increase in serum creatinine during ICU stay was observed in comparison to the patient’s baseline concentration; as “stage 2” if a 2 to 2.9-fold increase was observed; and “stage 3” if at least 3-fold increase was observed and/or implementation of any RRT technique required. The patient’s baseline serum creatinine concentration was estimated using the Modification of Diet in Renal Disease (MDRD) formula, as recommended by the Acute Dialysis Quality Initiative (ADQI) working group, assuming a glomerular filtration rate (GFR) equivalent to 75 mL/min as described in previous studies [10,11]. Total loss of renal function and the final AKI stage were defined according to the duration of the episode (>4 weeks) and/or the need for RRT during >3 months.

### 2.3. Data Collection

We collected the usual demographic, clinical, biological, management and outcome data from the patients’ records. Prolonged immobilization was considered if signs of compression (redness, blisters, pressure ulcers) were reported by the physician when examining the patient’s skin. Serum and urinary myoglobin concentrations were not collected as not routinely measured in our ICU. When several data were reported, the most critical values close to the ICU admission were considered.

### 2.4. Statistical Analysis

Data are expressed as the median (percentiles 25–75) or percentages as required. Variables were compared using Chi-2 or Fisher exact tests for the qualitative variables and Student t-tests for the quantitative variables. Variables significantly different at *p* < 0.2 were entered in multivariate logistic regression analyses with step-by-step selection procedure used to identify the variables independently associated with the risk of AKI, RRT requirement and death. For each quantitative variable, the analysis of the receiver operating characteristics (ROC) curve allowed the determination of the thresholds associated with the best sensitivity and specificity to predict the studied event (AKI, RRT or death). We calculated the odds ratio (OR) and their corresponding 95%-confidence intervals (CI). The performance of the predictive model was evaluated using the area under the curve (AUC) of the ROC curve (=1 for the optimal model and =0.5 for any model without a discriminating value). The statistical analyses were performed with XLAST^®^ software (Microsoft Excel, Microsoft Corporation, Redmond, WA, USA, 2016). The statistical significance was set at *p*-value < 0.05.

## 3. Results

### 3.1. Study Population

Two hundred and thirty-seven patients (138 men (58%)/99 women (42%); age, 41 years (31–53); new simplified acute physiology score (SAPSII) on admission, 33 (25–52)) were included. During the 7-year study period, 2418 poisoned patients were admitted to the ICU resulting in a prevalence of 9.8% for rhabdomyolysis in poisonings admitted to the ICU. The population characteristics are described in Table 1. Comorbidities were observed in 171 patients (72%). Attempted suicide by self-poisoning was reported in 222 patients (94%).

**Figure 1:**
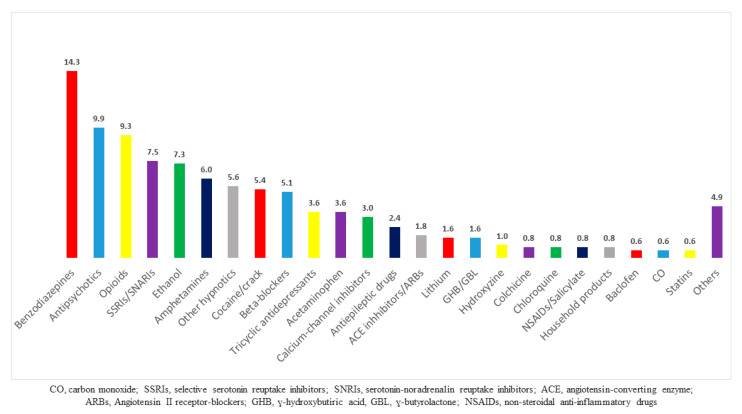
Toxicants involved in the poisonings with rhabdomyolysis based on the medical history (with their respective percentages noted above the bars).

Toxicants involved in the poisonings leading to rhabdomyolysis were pharmaceuticals (77%), drugs of abuse including ethanol (21%), household products (0.8%) and carbon monoxide (0.6%; Figure 1). The majority of poisonings were multidrug ingestions (63%) mainly involving psychotropic drugs and ethanol (blood ethanol concentration on admission, 1.13 g/L (0.29–2.01)). Traditional risk factors for rhabdomyolysis excluding possible toxicant-related direct toxicity were observed in 78 patients (33%) including seizures (18%), prolonged immobilization (16%) and falls (4%). Four cases of neuroleptic malignant syndrome were reported.

### 3.2. Features, Complications and General Management

Features on admission are summarized in Table 2. Complications at any point of ICU stay included aspiration pneumonia (54%), AKI (37%), cardiovascular failure (35%), cardiac arrest (14%), disseminated intravascular coagulation (12%), hospital-acquired infections (8%) and compartment syndrome (2%). Management included fluids (100%), antibiotics (61%), mechanical ventilation (52%), catecholamines (35%), 1.4% sodium bicarbonate (19%), RRT (18%) and extracorporeal membrane oxygenation (11%).

### 3.3. AKI Onset

**Figure 2:**
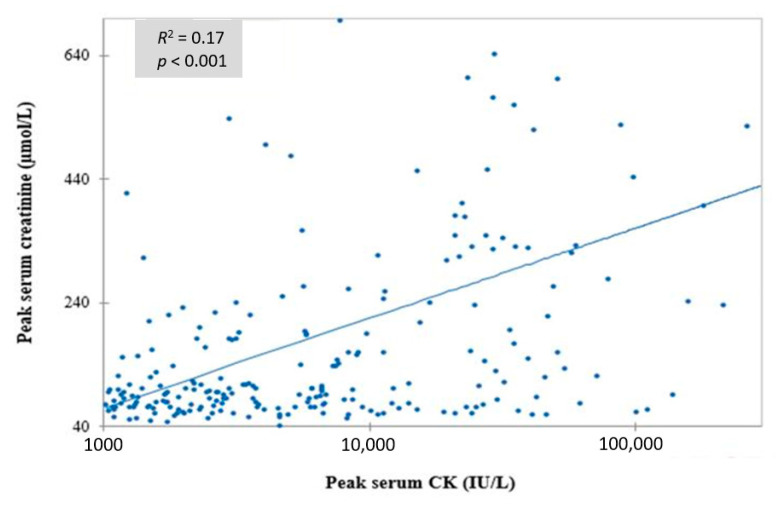
Correlation between the peak serum creatine kinase (CK) and the peak serum creatinine in the poisoned patients who developed rhabdomyolysis.

On ICU admission, serum CK and creatinine were 1,912 IU/L (624–6769) and 92 μmol/L (68–149), respectively. Peak CK and creatinine occurred 2 days (1–4) and 2 days (1–5) days after exposure, respectively, and were weakly correlated (linear regression; *R*^2^ = 0.17, *p* < 0.001) (Figure 2). According to the KDIGO classification, 88 patients (37%) developed AKI including stage 1 (N = 16, 18%), stage 2 (N = 14, 16%) and stage 3 (N = 58, 66%). No definitive loss of renal function or terminal AKI was observed. Except for the compartment syndrome, all complications were significantly more frequent in patients with AKI (Table 3).

**Figure 3:**
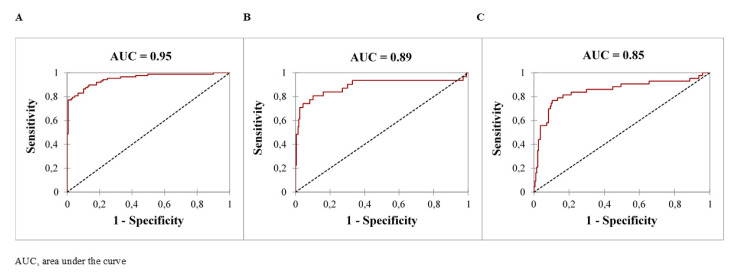
ROC curve of the different predictive models of acute kidney injury onset (**A**), renal replacement therapy requirement (**B**) and death (**C**) in the poisoned patients who developed rhabdomyolysis.

Based on univariate comparisons, variables significantly associated with AKI are summarized in Table 4. Interestingly, overdoses with beta-blockers, calcium-channel inhibitors, acetaminophen, colchicine, lithium, angiotensin-converting enzyme (ACE) and inhibitors/angiotensin II-receptor-blockers were significantly associated with an increased risk of AKI in our poisoned patients with rhabdomyolysis. By contrast, benzodiazepines and tricyclic antidepressant overdoses were significantly associated with a decreased risk. Using a multivariate logistic regression analysis (Table 5), lithium overdose (OR, 44.4; CI, 5.3–371.5), hypocalcemia with serum calcium ≤2.1 mmol/L (OR, 14.3; CI, 2.04–112.4), female gender (OR, 5.5; CI, 1.8–16.9), hyperphosphotemia with serum phosphate ≥1.5 mmol/L (OR, 2.0; CI, 1.0–4.2), serum lactate ≥3.3 mmol/L (OR, 1.2; IC, 1.1–1.4), serum creatinine ≥125 µmol/L (OR, 1.05; IC, 1.03–1.06) and patient age (OR, 1.04; IC, 1.01–1.07) were the independent variables on ICU admission significantly associated with AKI onset. The performance of our predictive model was supported by a ROC curve AUC of 0.95 (Figure 3A). Serum creatinine ≥ 125 μmol/L was the variable with the highest predictive value (AUC = 0.87), sensitivity (0.71) and specificity (0.92) for AKI onset.

### 3.4. RTT Requirement

Forty-three patients (49% of those who developed AKI, 18% of those who developed rhabdomyolysis) required RRT including hemodialysis (N = 29) and veno-venous continuous hemodiafiltration (N = 14). RTT was initiated 2 days (1–3) after ICU admission. Based on univariate comparisons, the variables significantly associated with RRT requirement are summarized in Table 6. Using a multivariate logistic regression analysis (Table 7), calcium-channel blocker overdose (OR, 14.2; CI, 3.8–53.6), serum phosphate ≥ 2.3 mmol/L (OR, 1.6; CI, 1.1–2.6), Glasgow coma score ≤ 5 (OR, 1.12; CI, 1.02–1.25), serum creatinine ≥ 125 µmol/L (OR, 1.01; CI, 1.00–1.01) and prothrombin index ≤ 71% (OR, 1.03; CI, 1.01–1.05) were the independent variables on ICU admission significantly associated with RRT requirement. The performance of our predictive model was supported by a ROC curve AUC of 0.89 (Figure 3B). Prothombin index ≤ 71% was the variable with the highest predictive value (AUC = 0.76) and sensitivity (0.70) for RRT. Serum phosphate ≥ 2.3 mmol/L was the variable with the highest specificity (0.95).

### 3.5. Additional Complications and Fatal Outcome

Thirty-one patients (13% of patients who developed rhabdomyolysis) died in the ICU. Mortality was significantly higher in patients with AKI (32% vs. 2%, *p* < 0.001). The main causes of death included multiorgan failure (65%), refractory cardiac arrest (19%), refractory cardiovascular failure (13%) and postanoxic encephalopathy (3%). The risk factors for death are summarized in Table 8. Using a multivariate logistic regression analysis (Table 9), KDIGO stage 3 AKI (OR, 7.0; CI, 2.5–19.8), serum phosphate ≥3.1 mmol/L (OR, 1.2; CI, 1.1–1.3) and prothrombin index ≤ 68% (OR, 1.04; CI, 1.02–1.06) were the independent variables on admission significantly associated with the risk of death in the ICU. The performance of our predictive model was supported by a ROC curve AUC of 0.85 (Figure 3C).

## 4. Discussion

In the poisoned patient admitted to the ICU and developing rhabdomyolysis, we established that AKI is a frequent complication, often requiring RTT and significantly associated with an increased risk of death. On admission, serum creatinine ≥125 μmol/L but not serum CK is the best predictor of AKI onset and RTT requirement.

Rhabdomyolysis diagnosis is based on serum CK. The classic triad (myalgia, transient muscle weakness and darker-colored urine) is inconsistently found (<10%) [2,12]. Electrolyte disturbances including hyperkalaemia, hyperphosphatemia, hypermagnesemia, hyperuricemia and metabolic acidosis occur, especially in the presence of AKI. Therefore, serum CK (mainly CK-MM isoenzymes) represent the most reliable and sensitive indicator of muscle damage [9,13]. Elevation in serum and/or urinary myoglobin is not a sensitive diagnostic criterion, thus infrequently performed in practice [2,14,15].

More than 150 toxicants are reported to lead to rhabdomyolysis; however, drugs of abuse, ethanol, statins, serotonin reuptake inhibitors and antiretroviral drugs are most commonly reported [1,2,3,12]. In our study, benzodiazepines (14%), antipsychotics (10%) and opioids (9%) were frequently involved, consistent with other reports [16,17,18]. Interestingly, four cases of neuroleptic malignant syndrome involving loxapine (N = 3) and olanzapine (N = 1) were observed. Serotonin reuptake inhibitors (N = 38) induced seizures (N = 12) and serotonin syndrome (N = 8). Illicit amphetamine-like stimulants (N = 30; mainly MDMA) involved seizures (N = 9) and malignant hyperthermia (N = 7), with marked severity as is well-recognized [19] and particular insight into the increased risk of severe rhabdomyolysis associated with synthetic cathinones has been discussed [20]. Finally, rhabdomyolysis was attributed to ethanol in the absence of coingestions in five patients (blood ethanol, 3.15 g/L (2.8–4.1)).

The primary rhabdomyolysis-induced complication is AKI. AKI is explained by tubule obstruction, reactive oxygen derivative production and renal arteriole vasoconstriction [1,2,7]. Following rhabdomyolysis, myoglobin precipitates in the renal tubules, decreasing urinary flow. The iron contained in the heme molecule of myoglobin produces free radicals resulting in cell membrane lipid peroxidation. The release of vasoactive molecules such as platelet activating factors, endothelin and prostaglandin F2α induces vasoconstriction of the renal arterioles and contributes to the decrease in glomerular filtration. Therefore, recommendations to lower the risk of AKI in patients with rhabdomyolysis include fluids to correct hypovolemia, achieve adequate diuresis and even dilute the released toxic endogenous metabolites, despite their relatively low level of evidence [1,2,3].

AKI occurred in 37.1% of our poisoned patients with rhabdomyolysis, in agreement with most of the previous studies limited [16,17,18] or not limited to poisonings [13,21]. Nevertheless, some studies reported higher incidence of AKI (up to 93%), probably due to different patient populations (e.g., elderly) and/or AKI definition [22,23]. Mortality was ~16 times higher in patients with AKI in comparison to those without AKI (31.8% vs. 2.0%, *p* < 0.001). Interestingly, our mortality rate was about 5–6 times higher than those previously reported in rhabdomyolysis patients [22,24,25], probably due to the recruitment of more severely ill patients and highly lethal poisonings such as colchicine (N = 4).

The predictive factors for AKI, RRT and death in rhabdomyolysis patients have been investigated previously; however, the studies did not specifically address the poisoned patient. The age, female gender, cause of rhabdomyolysis, serum creatinine, CK, bicarbonate, phosphate and calcium were generally identified as predictors of AKI, allowing the calculation of a predictive risk score [25]. Based on a mathematical equation using serum potassium, creatinine and albumin, rhabdomyolysis patients were also classified at “high” vs. “low risk” of AKI, although the formula was only validated in patients with serum creatinine < 265 μmol/L [13]. In our study, lithium overdose, age, female gender, elevated serum creatinine, phosphate, calcium and lactate were the independent predictors of AKI. All these variables yielded a ROC curve with an excellent predictive value. In another study, hypoalbuminaemia, metabolic acidosis, decline in prothrombin time and peak CK > 12,750 IU/L were predictive of AKI [24]; however, the predictive value of the model was lower than ours (AUC = 0.87 vs. 0.95, respectively). Lithium intoxication is responsible for functional renal impairment and tubular necrosis, independently of rhabdomyolysis [26]. Age was previously reported as predictive of AKI in rhabdomyolysis patients [4,25,27,28,29]. Glomerular filtration and renal blood flow are known to decrease by ~0.8% and ~1% per year by the age of 40, respectively [30]. Other age-related comorbidities such as hypertension, type-II diabetes and atherosclerosis likely contribute to the increased risk of AKI [31]. In our study, serum creatinine ≥ 125 μmol/L on admission was the highest predictive variable for AKI (AUC = 0.87) and RRT requirement (AUC = 0.85), consistent with previous studies [13,24,25,32,33]. It is obvious that creatinine is the main biomarker of AKI, widely used in clinical practice; however, due to its presence in large amounts in the myocyte, it is also an early marker of muscle lysis [1,13]. Hyperphosphatemia and hypocalcaemia are also related to muscle damage severity and renal function. Phosphate ions released during rhabdomyolysis together with their altered renal elimination induce tissue calcium salt precipitation causing hypocalcaemia [34]. Additional factors such as interference in vitamin D synthesis and resistance to parathyroid hormone may contribute to decrease serum calcium [35]. Interestingly, serum phosphate ≥3.1 mmol/L was also the variable with the highest predictive value of death (AUC = 0.85) in our study. Elevation in serum lactate on admission was significantly associated with the increased risk of developing AKI (OR = 1.2). There are two possible explanations: First, hyperlactatemia is a well-recognized biomarker of microcirculation impairment, thus explaining its accuracy in predicting AKI. Secondly, the massive release into the bloodstream during rhabdomyolysis of amino acids such as alanine may have led to pyruvate production by deamination and then to lactate under the action of lactate dehydrogenase [36].

In our study, calcium-channel inhibitor overdose, hyperphosphoremia, elevated serum creatinine, decreased prothrombin index and coma depth were the independent predictors of RTT requirement. Calcium-channel inhibitor overdoses are responsible for life-threatening cardiovascular failure and thus probably for more severe AKI requiring RRT (OR = 14.2). Low prothrombin index predicted both RRT requirement (OR = 1.03) and mortality (OR = 1.04). A previous study reported liver damage in ~25% of rhabdomyolysis patients [36]. Liver dysfunction is multifactorial resulting from hypotension and hyperthermia or related to the muscle release of proteases [37]. Patients with deep coma (Glasgow coma score ≤ 5) presented a greater need for RRT, as previously observed in head trauma patients [38]. We hypothesized that coma depth on admission is a good indicator of muscle compression/ischemia and thus of the severity of the resulting AKI. Finally, we found that stage-3 AKI, hyperphosphoremia and prothrombin index independently predicted death. In the ICU patients, AKI has consistently been associated with a significant increased risk of death [39].

We found a weak correlation between peak CK and peak creatinine (Figure 2) as previously observed [3,16]. Elevated CK concentration on admission was associated in our study with neither an increased risk of AKI nor RRT requirement, consistent with another study conducted in poisoned patients [18] but in contrast with the majority of the studies not focused on poisonings [3,5,24,29]. Such differences could be explained by the wide spectrum of nontoxic rhabdomyolysis etiologies which can elevate CK much higher than poisoning. Conformingly, CK > 5000 IU/L was present in 42.6% of septic patients versus 5.6% of poisoned patients (*p* < 0.0001) [40]. Large increases in CK have been reported after intense exercise without renal damage [41,42]. Underlying individual vulnerability related to gene polymorphism may also explain elevated CK levels after muscle damage [43]. Nevertheless, our results were comparable with other studies conducted in nonpoisoned patients, which did not demonstrate an association between high CK and AKI risk and reached the same conclusion that serum creatinine on admission is the best predictor of AKI [32,44]. The CK cut-off resulting in a higher risk of AKI, varied from 773 to 40,000 IU/L according to the studies [17,25,40], thus suggesting that CK is not a reliable prognostic criterion.

Our work presents limitations, mainly due to its retrospective methodology and the actual missing baseline serum creatinine that we had to estimate using the MDRD formula. By choosing the CK threshold of 1000 IU/L to define rhabdomyolysis, we may not have included some patients who actually presented muscle injury. We only relied on history and compatible features for some toxicants such as ACE inhibitors or angiotensin II receptor-blockers to analyze their involvement in the rhabdomyolysis due to the absence of available serum concentrations. AKI could not be considered as a single consequence of rhabdomyolysis in our severely poisoned patients with possible other concomitant causes of AKI such as sepsis and nephrotoxic drug administration (i.e., aminoglycosides or glycopeptides to treat infections and iodine for imaging purposes). Finally, rare patients were treated with RRT to enhance the toxicant clearance independently of AKI, resulting in possible overestimation of KDIGO stage-3 patients. All our poisoned patients treated with RRT left the ICU once not requiring additional RRT sessions but were transferred to the medical or psychiatric wards before complete normalization of their serum creatinine, thus precluding determining the exact time to renal recovery.

## 5. Conclusions

Poisoned patients presenting with rhabdomyolysis are at elevated risk of AKI and consequently increased risk of ICU mortality. Serum creatinine ≥ 125 μmol/L on admission is the best predictor of AKI, whereas serum CK is not. Our findings could help clinicians identify patients with rhabdomyolysis at higher risk of developing AKI in order to rapidly implement adequate monitoring and optimal life-saving management. Prospective studies on larger cohorts remain required to demonstrate the relevance of our findings to improving patient prognosis.

## Figures and Tables

**Table 1:** Characteristics of the study population.

Parameters	Median (Percentiles 25–75) or N (%)
Demographics	
Age (years)	41 (31–53)
Body mass index (kg/m^2^)	23.6 (21.0–27.2)
Gender (Male/Female)	138 (58%)/99 (42%)
Ethnicity	
Caucasian	181 (76%)
North-African	30 (13%)
Black African	18 (8%)
Asian	5 (2%)
Indian	3 (1%)
Exposure	
Intentional/Accidental	222 (94%)/15 (6%)
Time from exposure to admission	
<4 h	63 (29%)
4–8 h	64 (29%)
8–24 h	67 (31%)
>24 h	26 (12%)
Medical history	
Past depression/suicide attempt	192 (81%)
Drug addiction	87 (37%)
Chronic alcoholism	65 (27%)
Psychosis	56 (24%)
Hypertension/coronary disease	32 (14%)
Human immunodeficiency virus infection	19 (8%)
Diabetes/dyslipidemia	16 (7%)
Long-term statin treatment	9 (4%)
Number of long-term medications	3 (1–5)

**Table 2:** Clinical features of the poisoned patients with rhabdomyolysis on admission to the intensive care unit.

Parameters	Median (Percentiles 25–75) or N (%)
Glasgow coma score	8 (3–14)
Systolic blood pressure (mmHg)	116 (101–135)
Diastolic blood pressure (mmHg)	72 (58–83)
Heart rate (/min)	93 (75–105)
Respiratory rate (cycles/min)	17 (14–21)
Normopnea (12–20/min)	134 (58%)
Tachypnea (>20/min)	80 (35%)
Bradypnea (<12/min)	18 (8%)
Agitation/confusion	73 (31%)
Nausea/vomiting	45 (19%)
Oliguria/anuria	38 (16%)

**Table 3:** Complications in the intensive care unit poisoned patients who developed rhabdomyolysis with vs. without acute kidney injury (AKI).

Complications	Patients without AKI N (%)	Patients with AKI N (%)	*p*-Value
Infections	77 (52%)	67 (76%)	0.001
DIC	3 (2%)	26 (30%)	<0.0001
Cardiac arrest	4 (3%)	28 (32%)	<0.0001
Cardiovascular failure	21 (14%)	62 (71%)	<0.0001
Compartment syndrome	1 (0.7%)	4 (5%)	0.07

DIC, disseminated intravascular coagulation.

**Table 4:** Clinical and laboratory parameters in the poisoned patients who developed rhabdomyolysis according to the onset of acute kidney injury (AKI).

Characteristics	Without AKI (N = 149)	With AKI (N = 88)	*p*-Value
Demographics			
Age (years)	39 (30–50)	49 (34–55)	0.005
Female gender	57 (38%)	42 (48%)	0.2
Delay exposure/admission (≥24 h)	11 (7%)	22 (25%)	0.001
Past history of hypertension	10 (7%)	22 (25%)	<0.0001
Long-term treatment with statins	3 (2%)	6 (7%)	0.06
Involved toxicants			
Benzodiazepines	52 (34.9)	20 (23%)	0.05
Tricyclic antidepressants	16 (11%)	2 (2%)	0.02
Beta-blockers	11 (7%)	15 (17%)	0.02
Acetaminophen	6 (4%)	12 (14%)	0.007
Calcium-channel inhibitors	4 (3%)	11 (13%)	0.003
ACE/Angiotensin II receptor-blockers	2 (1%)	7 (8%)	0.01
Lithium	3 (2%)	5 (6%)	0.1
Colchicine	0 (0)	4 (4%)	0.009
Clinical parameters			
Glasgow coma score	9 (4–14)	6 (3–12)	0.005
Laboratory parameters			
Serum creatinine (µmol/L)	75 (62–95)	160 (104–246)	<0.0001
Blood urea nitrogen (mmol/L)	5.2 (4.1–6.6)	7.8 (6.1–13.3)	<0.0001
Serum creatine kinase (IU/L)	1935 (696–5826)	1833 (265–8834)	0.06
Arterial pH	7.39 (7.32–7.44)	7.29 (7.23–7.40)	<0.0001
Serum bicarbonate (mmol/L)	22 (20–25)	19 (14–23)	<0.0001
Serum lactate (mmol/L)	1.6 (0.9–3.0)	4.1 (1.9–8.7)	<0.0001
Serum potassium (mmol/L)	3.9 (3.6–4.3)	4 (3.4–4.6)	0.08
Serum calcium (mmol/L)	2.2 (2.1–2.3)	2.0 (1.8–2.2)	<0.0001
Serum phosphate (mmol/L)	1.1 (0.9–1.3)	1.5 (1.1–2.4)	<0.0001
ALT (IU/L)	37 (24–71)	88 (35–197)	<0.0001
AST (IU/L)	82 (44–164)	198 (77–572)	0.001
Serum lactate dehydrogenase (IU/L)	324 (240–459)	652 (354–1851)	<0.0001
Prothrombin index (%) *	88 (75–96)	67 (47–81)	<0.0001
Platelets (G/L)	242 (184–282)	221 (165–283)	0.2
Serum albumin (g/L)	35 (30–38)	24 (20–32)	<0.0001

ACE, angiotensin-converting enzyme; ALT, alanine aminotransferase; AST, aspartate aminotransferase; * the prothrombin index is the ratio of the normal prothrombin time to the patient’s prothrombin time, expressed as a percentage.

**Table 5:** Variables on intensive care unit admission significantly associated with the risk of acute kidney injury in the poisoned patient with rhabdomyolysis based on a multivariate logistic regression analysis.

Variables	Odds Ratio [95%-IC]	Sensitivity [95%-IC]	Specificity [95%-IC]	Positive Predictive Value [95%-IC]	Negative Predictive Value [95%-IC]	Accuracy [95%-IC]	AUC ROC Curve [95%-IC]	*p-*value
Lithium overdose	44.4 [5.3–371.5]							0.001
Serum calcium ≤2.1 mmol/L	14.3 [2.04–112.4]	0.58 [0.48–0.68]	0.79 [0.72–0.85]	0.62 [0.52–0.72]	0.76 [0.69–0.83]	0.71 [0.61–0.81]	0.70 [0.61–0.79]	0.01
Female gender	5.5 [1.8–16.9]							0.003
Serum phosphate ≥1.5 mmol/L	2.0 [1.0–4.2]	0.52 [0.42–0.63]	0.85 [0.78–0.90]	0.67 [0.57–0.77]	0.75 [0.66–0.82]	0.73 [0.64–0.82]	0.68 [0.59–0.77]	0.05
Serum lactate ≥3.3 mmol/L	1.2 [1.1–1.4]	0.64 [0.54–0.74]	0.79 [0.72–0.85]	0.65 [0.55–0.75]	0.79 [0.70–0.88]	0.74 [0.65–0.83]	0.75 [0.68–0.83]	0.002
Serum creatinine ≥125 µmol/L	1.05 [1.03–1.06]	0.71 [0.60–0.79]	0.92 [0.86–0.95]	0.84 [0.76–0.92]	0.84 [0.76–0.92]	0.84 [0.76–0.92]	0.87 [0.81–0.93]	<0.0001
Age ≥48 years	1.04 [1.01–1.07]	0.52 [0.42–0.62]	0.71 [0.63–0.77]	0.51 [0.41–0.61]	0.71 [0.62–0.80]	0.64 [0.54–0.74]	0.61 [0.53–0.70]	0.005

95%-IC: 95%-confidence interval; AUC: area under curve; ROC: receiver operating characteristics.

**Table 6:** Clinical and laboratory parameters in the poisoned patients who developed rhabdomyolysis according to renal replacement therapy (RTT) requirement.

Characteristics	Without RRT (N = 194)		With RRT (N = 43)		*p*-Value	
Past history of hypertension	23 (11.9)		9 (20.9)		0.1	
Involved toxicants						
Benzodiazepines	64 (33%)		8 (19%)		0.06	
Antipsychotics	45 (23%)		5 (12%)		0.09	
Calcium-channel inhibitors	6 (3%)		9 (21%)		0.001	
Chloroquine	2 (1%)		2 (5%)		0.1	
Colchicine	0 (0%)		4 (9%)		<0.0001	
Clinical parameters						
Glasgow coma score	9 (4–14)		3 (3–11)		0.002	
Laboratory parameters						
Serum creatinine (µmol/L)	86 (65–120)		155 (95–246)		<0.0001	
Blood urea nitrogen (mmol/L)	5.7 (4.3–7.7)		6.9 (5.7–11.6)		0.01	
Serum creatine kinase (IU/L)	2070 (633–6788)		1676 (203–4935)		0.04	
Arterial pH	7.38 (7.31–7.43)		7.26 (7.18–7.40)		<0.0001	
Serum bicarbonate (mmol/L)	22 (19–25)		18 (14–22)		<0.0001	
Serum lactate (mmol/L)	1.7 (1.0–3.8)		4.5 (3.0–9.4)		<0.0001	
Serum calcium (mmol/L)	2.2 (2.1–2.3)		2.0 (1.7–2.2)		0.001	
Serum phosphate (mmol/L)	1.1 (0.9–1.4)		1.5 (1.1–2.9)		<0.0001	
Serum ALT (IU/L)	42 (25–90)		99 (36–232)		0.02	
Serum AST (IU/L)	101 (46–213)		207 (74–615)		0.0057	
Serum lactate dehydrogenase (IU/L)	363 (257–606)		789 (353–2255)		<0.0001	
Red blood cell count (T/L)	4.44 (4.02–4.91)		4.36 (3.63–4.60)		0.01	
Hemoglobin (g/dL)	13.6 (12.6–15.1)		13.3 (11.5–14.5)		0.04	
Prothrombin index (%) *	83 (71–95)		60 (43–76)		<0.0001	
Serum albumin (g/L)	34 (28–38)		24 (20–32)		<0.0001	

ALT: alanine aminotransferase; AST: aspartate aminotransferase; * the prothrombin index is the ratio of the normal prothrombin time to the patient’s prothrombin time, expressed as a percentage.

**Table 7:** Variables on intensive care unit admission significantly associated with the requirement of renal replacement therapy in the poisoned patient with rhabdomyolysis based on a multivariate logistic regression analysis.

Variables	OR [95%-IC]	Sensitivity [95%-IC]	Specificity [95%-IC]	Predictive Positive Value [95%-IC]	Predictive Negative Value [95%-IC]	Accuracy [95%-IC]	AUC ROC Curve [95%-IC]	*p*-Value
Calcium-channel blocker overdose	14.2 [3.8–53.6]							<0.0001
Serum phosphate ≥2.3 mmol/L	1.6 [1.1–2.6]	0.43 [0.29–0.58]	0.95 [0.91–0.98]	0.67 [0.53–0.81]	0.88 [0.78–0.98]	0.86 [0.76–0.96]	0.67 [0.55–0.80]	0.03
Glasgow coma score ≤5	1.12 [1.02–1.25]	0.63 [0.48–0.76]	0.71 [0.64–0.77]	0.33 [0.19–0.47]	0.90 [0.81–0.99]	0.70 [0.56–0.84]	0.66 [0.55–0.76]	0.02
Prothrombin index * ≤71%	1.03 [1.01–1.05]	0.70 [0.55–0.81]	0.74 [0.67–0.80]	0.38 [0.23–0.53]	0.92 [0.86–0.98]	0.73 [0.60–0.86]	0.76 [0.66–0.86]	0.001
Serum creatinine ≥125 µmol/L	1.01 [1.00–1.01]	0.65 [0.50–0.78]	0.76 [0.70–0.82]	0.38 [0.23–0.53]	0.91 [0.82–0.99]	0.74 [0.61–0.87]	0.75 [0.66–0.84]	0.02

95%-IC, 95%-confidence interval; AUC, area under curve; ROC, receiver operating characteristic; * the prothrombin index is the ratio of the normal prothrombin time to the patient’s prothrombin time, expressed as a percentage.

**Table 8:** Clinical and laboratory parameters in the poisoned patients who developed rhabdomyolysis according to survival in the intensive care unit.

Characteristics	Survivors (N = 206)	Non-Survivors (N = 31)	*p*-Value
Age (years)	41 (31–53)	49 (34–57)	0.2
Involved toxicants			
Antipsychotics	49 (24%)	1 (3%)	0.001
Beta blockers	20 (10%)	6 (19%)	0.1
Paracetamol	13 (6%)	5 (16%)	0.06
Calcium-channel blockers	10 (5%)	5 (16%)	0.01
Chloroquine	2 (1%)	2 (7%)	0.03
Colchicine	1 (1%)	3 (10%)	0.001
Clinical parameters			
Glasgow coma score	9 (3–14)	3 (3–8)	0.001
Temperature (°C)	36.8 (35.8–37.9)	35.7 (34.1–36.2)	0.1
AKI (stage 3, KDIGO)	35 (17%)	23 (74%)	<0.0001
Laboratory parameters			
Serum creatinine (µmol/L)	88 (66–127)	147 (95–243)	<0.0001
Blood urea nitrogen (mmol/L)	5.9 (4.3–7.7)	6.9 (5.4–11.6)	0.001
Arterial pH	7.38 (7.30–7.43)	7.24 (7.12–7.40)	< 0.0001
Serum bicarbonate (mmol/L)	22 (19–25)	17 (13–21)	< 0.0001
Serum lactate (mmol/L)	1.8 (1.0–3.9)	8.2 (3.6–12.3)	<0.0001
Serum calcium (mmol/L)	2.2 (2.1–2.3)	1.9 (1.7–2.1)	<0.001
Serum phosphate (mmol/L)	1.1 (0.9–1.5)	2.0 (1.2–3.1)	<0.0001
Serum ALT (UI/L)	43 (24–91)	157 (54–531)	<0.0001
Serum AST (UI/L)	101 (47–213)	332 (111–900)	0.001
Serum lactate dehydrogenase (UI/L)	371 (257–630)	859 (416–2875)	0.001
Hemoglobin (g/dL)	13.6 (12.6–15.1)	12.5 (11.0–14.3)	0.003
Platelets (G/L)	236 (184–281)	209 (149–286)	0.1
Prothrombin time (%) *	83 (71–94)	47 (30–63)	<0.0001

AKI: acute kidney injury; KDIGO: kidney disease improving global outcomes; ALT: alanine aminotransferase; AST: aspartate aminotransferase; * the prothrombin index is the ratio of the normal prothrombin time to the patient’s prothrombin time, expressed as a percentage.

**Table 9:** Predictive factors of death on intensive care unit admission in the poisoned patient with rhabdomyolysis based on a multivariate logistic regression analysis.

Variables	OR [95%-IC]	Sensitivity [95%-IC]	Specificity [95%-IC]	Predictive Positive Value [95%-IC]	Predictive Negative Value [95%-IC]	Accuracy [95%-IC]	AUC ROC Curve [95%-IC]	*p*-Value
Acute kidney injury, stage 3 According to the KDIGO classification	7.0 [2.5–19.8]							0.001
Hyperphosphoremia ≥3.1 mmol/L	1.2 [1.1–1.3]	0.84 [0.67–0.93]	0.81 [0.75–0.86]	0.40 [0.28–0.52]	0.97 [0.94–0.99]	0.81 [0.76–0.86]	0.85 [0.80–0.92]	0.001
Prothrombin index * ≤68%	1.04 [1.02–1.06]	0.84 [0.67–0.93]	0.70 [0.62–0.75]	0.30 [0.19–0.41]	0.97 [0.94–0.99]	0.71 [0.65–0.77]	0.83 [0.72–0.95]	0.001

95%-IC: 95%-confidence interval; AUC: area under curve; ROC: receiver operating characteristic; * the prothrombin index is the ratio of the normal prothrombin time to the patient’s prothrombin time, expressed as a percentage.
